# Supplementary material for: Determinants of individuals’ belief in fake news: A scoping review determinants of belief in fake news
Source: PLoS One. 2021 Jun 24;16(6):e0253717. doi: 10.1371/journal.pone.0253717 (PMC8224890; doi:10.1371/journal.pone.0253717)
Supplement: Attachment — Submitted filename: Response to reviewers.docx [file pone.0253717.s001.docx]

**Rebuttal letter**

Dear Drs. Altay, Lewandowsky, and Yesilada,

We are extremely grateful for your insightful and generously detailed feedback to our work. Based on your comments and suggestions, we have introduced some major changes to our report’s structure and evidence presentation. We believe that what resulted from this collaborative effort is a significantly improved manuscript. Our detailed responses to your comments, in the order we have received them, are listed in the table below. We hope that you will find these responses sufficient.

Kind regards,

The authors.

| **Reviewer** | **Comment** | **Response** |
| --- | --- | --- |
| R1 | P24: “In sum, findings in this section suggest that the general warnings and non-specific rhetoric of “fake news” should be employed with caution so as to avoid the possible backfire effect.” ---> What do the authors mean by backfire effect? It is used in the literature to mean a lot of different things. | We revised the paragraph that discusses the possibility of backfire effects (page 32, lines 689–697) to clarify the meaning of the term and reflect, following the advice of Drs. Yesilada and Lewandowsky, the recent findings that call the empirical robustness of the phenomenon into question. |
| R1 | P26: “The line of research concerned with accuracy-improving interventions reveals limited efficiency of general warnings and Facebook-style tags and suggests that simple interventions embedded in news interfaces to prime critical thinking and exposure to news literacy guidelines can induce more reliable improvements while avoiding normatively undesirable backfire effects.” ---> Add commas (e.g. after “tags”) otherwise there is no space to breathe. | The sentence was revised (page 34, lines 743–746). |
| R1 | The “truth bias” is, I believe, psychologically implausible (Sperber, D., Clément, F., Heintz, C., Mascaro, O., Mercier, H., Origgi, G., & Wilson, D. (2010). Epistemic vigilance. Mind & Language, 25(4), 359-393). However, it is, for some reason, very influential in the literature on misinformation. The authors’ result regarding the absence of truth bias could be mentioned in the discussion. Indeed, this implausible assumption could influence the way researchers design their experiments and frame their results.  On the other hand, the “deception bias” makes sense in light of what we know about trust in the digital age: the problem is not that people trust fake news sources too much but rather that they don’t trust good sources enough (e.g. Fletcher, R., & Nielsen, R. K. (2019); Altay, S., Hacquin, AS. & Mercier, H. (2020); Pennycook, G., & Rand, D. G. (2019); Mercier, H. (2020). | We added a paragraph to the Discussion section (page 33, lines 713–720) reflecting on the possibility that the “deception bias” is increasingly prevalent and can be problematic as well. |
| R2 | Major point: The number of studies included in the scoping review that investigate fake news interventions was limited. The intervention section highlights the prominence of inoculation-based research in this context; however, we noticed some studies that could have been included but that were not (e.g., Basol, Roozenbeek, & van der Linden, 2020; Roozenbeek & van der Linden, 2019). These studies should be included to provide a comprehensive overview of the research area. | In response to Drs. Yesilada and Lewandowsky's comment regarding certain relevant studies missing from our review, we audited our search protocol to understand why these studies were not included in the original pool. We found that Roozenbeek & van der Linden, 2019, was filtered out on the stage of full-text assessment for eligibility (please see our selection workflow chart for details) on the grounds of not fully meeting the true experimental design criterion: The experiment in question utilized within-subject measurement of the dependent variable and did not include a randomized control group. Basol, Roozenbeek, & van der Linden, 2020, which similarly tested the effects of playing the game *Bad News* on people's ability to spot misinformation, and whose design included a control group, was published in the Journal of Cognition. This scholarly publication that was indexed in neither Scopus or Web of Science at the time of our search, and therefore could not appear in the sample.  Given clear theoretical importance of these studies, we decided to include them into our review by doing the following: 1) Relaxing the requirement of necessarily having a randomized control group in the study design, which led to reinstating Roozenbeek & van der Linden, 2019 to the sample of studies we analyzed. Following this modification, we went back to the full-text assessment stage of our selection process and found that Roozenbeek & van der Linden, 2019 was the only work excluded based on this criterion. 2) Given that the design of Basol, Roozenbeek, & van der Linden, 2020, remedies some of the shortcomings of the original test's design, we mention their findings in parts of Results and Discussion sections that discuss inoculation interventions, albeit we do not include this study in the main sample.  Furthermore, reviewer feedback regarding insufficient coverage of the relevant literature on inoculation-based interventions led us to modify our search protocol altogether by adding the following terms: resist* OR immun* or innocul*. As a result, compared to the original version of the manuscript, two new studies appeared in the sample in addition to Roozenbeek & van der Linden, 2019: 1) Amazeen & Bucy, 2019, and 2) Tsang, 2020 - based on its title, this article should have appeared in the results of the original search, yet for some reason it did not. |
| R2 | The discussion needs further organization into subsections to make it clear to the reader where to locate information. There is no real conclusion subsection which makes it difficult to tie together the report's implications and findings. Also, much of the methods section (page 7-9) seems to assess and evaluate the included studies' methodological decisions rather than describing the review's methodology. Although this information is valuable, it is perhaps better suited for the results or discussion section. | We took the following steps to rearrange the structure of the report:  1)    Data charting subsection was moved from Methods to Results (now begins with the sub-header ‘Outcome variables’ (page 9, line 270);  2)    Assessment of the included studies’ methodology was moved to the new section, ‘Conclusions and future research;’  3)    A new section, ‘Limitations,’ added to discuss the review’s own methodological limitations. |
| R2 | It is not entirely clear how the research team identified the three-factor groups (message-level factors, individual-level factors, and intervention factors). Were these groups based on a precedent, or is there consensus in the research area that factors can be categorized into these three groups? It is important to have this information to justify the methodology and determine if any potentially important factors were missed. Also, the intervention factor group is paired with ecological factors - it is not entirely clear what the research team means by "ecological factors". | Our original classification of factor groups was not underlain by any existing scholarly consensus regarding what factors belong together. Rather, it emerged a posteriori following our consideration of what variables the studies included in our sample investigate as potential sources of variation in individuals' likelihood to believe online misinformation.  It seemed logical to us that studies testing the effects of people's individual characteristics (such as cognitive style, emotionality, proneness to conspiracy thinking, etc.) are qualitatively different from those looking at factors outside of an individual recipient, namely, message characteristics and presentation cues. We concluded that these two streams of scholarship seek to address the questions at the core of our review from two distinct vantage points (Who believes fake news? vs What fake news are most believed?), hence the distinction.  By ecological (environmental) factors, we understand the features that describe a broader information environment where users encounter news content rather than a particular message - for example, the prevalence of elite 'fake news' rhetoric examined by van Duyn and Collier (2018). Initially, we lumped these studies together with the effects of interventions because both these groups cannot be attributed to neither individual nor message level. However, upon further consideration and receiving reviewer feedback we decided to revise the classification altogether. The new categorization runs as follows: 1) Individual factors (everything within message recipient); 2) Message-level and environmental factors; 3) Targeted interventions. |
| R2 | Table three, which sets out the key methodological aspects and results of the included studies, needs more procedural information. At present, it is not easy to interpret how the included studies might have arrived at their results. This information could provide a more comprehensive summary of the research area, particularly for people who might want to know more about the commonalities amongst procedures across studies. | To address this concern, in Table 3, we expanded the scope of the column previously entitled, ‘Stimulus,’ to include brief descriptions of the included studies’ procedures. There, we provided design statements specifying factor levels, and described the order of key variables’ measurement, stimuli, and other meaningful procedural details, as necessary. The modified column is now entitled, ‘Procedures and stimuli.’ |
| R2 | 2:1:3 The end of this sentence requires a citation. A study or report that has mapped out propaganda, misinformation, and deception in the public sphere over time would be relevant to cite here. | Citation added (page 2, line 51).  Gorbach, J. (2018). Not Your Grandpa’s Hoax: A Comparative History of Fake News. *American Journalism*, *35*(2), 236–249. <https://doi.org/10.1080/08821127.2018.1457915> |
| R2 | 2:1:6-7 The authors state, "a lack of consensus over the scale and persuasiveness of the phenomenon". However, it is not clear what they are referring to. We assume they are discussing the persuasiveness of misinformation in general. It is also unclear how the references they cited support such a claim. | The sentence was reworded, examples and following citations added (page 2, lines 54–59).   - Ognyanova K, Lazer D, Robertson R, Wilson C. Misinformation in action: Fake news exposure is linked to lower trust in media, higher trust in government when your side is in power. Harvard Kennedy Sch Misinformation Rev. 2020;1(4):1–19. - Balmas M. When Fake News Becomes Real: Combined Exposure to Multiple News Sources and Political Attitudes of Inefficacy, Alienation, and Cynicism. Communic Res. 2014;41(3). - Murphy G, Loftus EF, Hofstein Grady R, Levine LJ, Greene CM. Erratum: False Memories for Fake News During Ireland’s Abortion Referendum. Psychol Sci. 2019;30:1449–1459. - Jost PJ, Pünder J, Schulze-lohoff I. Fake news - Does perception matter more than the truth ? J Behav Exp Econ. 2020;85:101513. |
| R2 | 3:1:1-5 The authors refer to a "massive spread of politically charged deception". To a certain extent, the word massive is subjective and does not point to the problem's true extent. With that in mind, some statistics or figures would be helpful. | Sentence reworded (page 3, lines 71–74). Empirical example provided based on the citation:  Allcott H, Gentzkow M. Social Media and Fake News in the 2016 Election. J Econ Perspect. 2017; 31(2):211–36. |
| R2 | 3:1:7 The authors note the "hypothesized societal effects" of deceitful messages; however, they do not explain what these societal effects might be. There are some studies out there that have investigated the causal effects of misinformation. These studies might be a good idea to cite to set the scene for the study. See below for a selection:  Schaub, M., & Morisi, D. (2020). Voter mobilisation in the echo chamber: Broadband internet and the rise of populism in europe. European Journal of Political Research, 59(4), 752–773. https://doi.org/10.1111/1475-6765.12373  Bursztyn, L., Egorov, G., Enikolopov, R., & Petrova, M. (2019). Social media and xenophobia: evidence from Russia (No. w26567). National Bureau of Economic Research.  Allcott, H., Braghieri, L., Eichmeyer, S., & Gentzkow, M. (2020). The welfare effects of social media. American Economic Review, 110(3), 629–76. DOI: 10.1257/aer.20190658 | Examples of the effects provided based on the following citations (page 3, lines 81–82):   - Allcott BH, Braghieri L, Eichmeyer S, Gentzkow M. The Welfare Effects of Social Media. Am Econ Rev 2020,. 2020;110(3):629–76. - Tucker JA, Guess A, Barberá P, Vaccari C, Siegel A, Sanovich S, et al. Social Media, Political Polarization, and Political Disinformation: A Review of the Scientific Literature. 2018;1–95. Available from: <https://papers.ssrn.com/abstract=3144139> - Weeks BE. Emotions, Partisanship, and Misperceptions: How Anger and Anxiety Moderate the Effect of Partisan Bias on Susceptibility to Political Misinformation. J Commun. 2015;65:699–719. |
| R2 | 3:2:4 Citation needed to support this claim. | Citation provided, sentence reworded (page 3, lines 87–90).  Tandoc EC. The facts of fake news: A research review. Sociol Compass. 2019;13:1–9. |
| R2 | 3:2:6 The authors refer to a "focal issue", but it is not entirely clear what the focal issue is. | Sentence reworded (page 3, line 92). |
| R2 | 4:1:5 The Sentence started with "because", but because of what? Consider re-writing for clarity. | Sentence reworded (page 4, line 127). |
| R2 | 4:3:2 The authors use the term "inductively developed framework", it would be good if the authors described what this means. | The term was clarified (page 5, lines 138–143). In particular, we have explained what led us to the factor categorization presented in the results section. |
| R2 | 5 The eligibility criteria would benefit from being placed in a table. At present, the criteria are embedded in the text, and it does not make it easy for the reader to identify the information. | We have decided not to create a separate table with selection criteria because they are already visually presented in the flow diagram illustrating the process of study selection (Fig 1). |
| R2 | 5:1:2 The time frame for the included studies should be explained. We assume the time frame for the included studies starts in 2016 to coincide with the initial Trump presidential campaign, but this an assumption. Further clarification would help. | Time frame has been clarified (page 6, lines 158–161). |
| R2 | 5:1:7-12 This Sentence is far too long, consider rewording or breaking it down into several sentences. | Sentence reworded (page 6, 165–169). |
| R2 | 5:1:12-14 The search started from studies already known to the researchers; cite this here for clarity. | Citations added (page 6, line 171):   - Kim A, Dennis AR. Says who? The effects of presentation format and source rating on fake news in social media. Mis Q. 2019;43(3):1025–39. - Pennycook G, Rand DG. Lazy, not biased: Susceptibility to partisan fake news is better explained by lack of reasoning than by motivated reasoning. Cognition. 2019;188:39–50. - Van Duyn E, Collier J. Priming and Fake News: The Effects of Elite Discourse on Evaluations of News Media. Mass Commun Soc. 2019;22(1):29–48. - Guess AM, Nyhan B, Reifler J. Exposure to untrustworthy websites in the 2016 US election. Nat Hum Behav. 2020;4:472–480. |
| R2 | 5:2 This sentence is too long and would benefit from shortening. Also, the paragraph states that the trio of databases would most likely yield the most comprehensive results - but why? This needs to be clearly explained. | The explanation supporting the choice of databases had been provided (page 7, lines 196–204). |
| R2 | 6:3:4 The authors do not explain why they chose these outcome variables. | Clarification provided (page 8, 228–230). We specify that these variables were selected after potentially eligible articles had been screened. |
| R2 | 8:1:1 The authors wrote, "As visible from the table", but do not state which table they refer to. | Clarified (page 10, line 278). |
| R2 | 10:1 This paragraph would be a good place to explain how the factor groups were identified. | Detailed explanation of how the factor groups were identified has been provided (page 12–13, lines 320–336). |
| R2 | 10:2:1 Avoid using rhetorical questions. | Sentence reworded (page 13, 342–343). |
| R2 | 11:1:3-5 This Sentence is wordy and unclear; consider re-writing. | Sentence reworded (page 13, lines 350–352). |
| R2 | 11:2:10-12 It is unclear what these numbers mean concerning the scale – But why? | Sentence corrected (page 4, line 386–387). |
| R2 | 16:1:3 The authors note that "two major message level factors stand out"; however, it is not explained why these two stand out in particular. | Sentence reworded (page 22, lines 402–403). |
| R2 | 17:1:1-5 This sentence is too long, consider rewording or breaking it down into several sentences. | Sentence shortened (page 22, lines 416–419). |
| R2 | 17:1:7-10 This sentence is not clear on its own. Another sentence is needed to explain why these methodological differences lead to differing results. | Clarification added (page 23, lines 429–431). |
| R2 | 17:1:16-18 It is argued that the differences in findings might be down to different study design choices - however, this needs more unpacking. The sentence alone does not explain why. | Explanation added (page 23, lines 438–440). |
| R2 | 17:1:1-5 sentence is too long; consider rewording. | Sentence shortened (page 22, lines 416–419). |
| R2 | 19:2:2 The authors use the terms "vary dramatically"; however, it is unclear what this means exactly; some figures or further quantification would be handy here. | Sentence reworded (page 25, 490–493). |
| R2 | 19:2:8 The authors note an apparent limitation, but further explanation is needed to determine why it is a limitation. | Explanation provided (page 25, lines 498–499). |
| R2 | 19:3:3-4 Citation needed. | Following citations provided (page 26, line 515):   - Evans JSBT, Stanovich KE. Dual-Process Theories of Higher Cognition: Advancing the Debate. Perspect Psychol Sci. 2013;8(3):223 –241. - Pennycook G, Fugelsang JA, Koehler DJ. What makes us think? A three-stage dual-process model of analytic engagement. Cogn Psychol. 2015;80:34–72. |
| R2 | 19:3:4-6 Citation needed. | Citations provided (page 26, line 517):   - Bronstein MV, Pennycook G, Bear A, Rand DG, Cannon TD. Belief in Fake News is Associated with Delusionality, Dogmatism, Religious Fundamentalism, and Reduced Analytic Thinking. J Appl Res Mem Cogn. 2019;8(1):108–17. - Pennycook G, Rand DG. Who falls for fake news? The roles of bullshit receptivity, overclaiming, familiarity, and analytic thinking. J Pers. 2019;88:185–200. |
| R2 | 20:2:5-7 The authors state that the correlations are statistically significant but do not provide an indicator of significance. | Indicator of significance provided (page 26, line 532–533). |
| R2 | 23:1:2 Citation needed. | Following citations were added to the paragraph (page 29, lines 606, 608):   - Jones-Jang SM, Mortensen T, Liu J. Does Media Literacy Help Identification of Fake News? Information Literacy Helps, but Other Literacies Don’t. Am Behav Sci. 2019;1–18. - Roozenbeek J, van der Linden S. Fake news game confers psychological resistance against online misinformation. Palgrave Commun. 2019;5(65):1–10. - Clayton K, Blair S, Busam JA, Forstner S, Glance J, Green G, et al. Real Solutions for Fake News? Measuring the Effectiveness of General Warnings and Fact‑Check Tags in Reducing Belief in False Stories on Social Media. Polit Behav. 2019; - Garrett RK, Poulsen S. Flagging Facebook Falsehoods: Self-Identified Humor Warnings Outperform Fact Checker and Peer Warnings. J Comput Commun. 2019;24:240–58. |
| R2 | 23:2:2-5 Citation needed. | Following citations provided (page 30, lines 612–619):   - Jolley D, Douglas KM. Prevention is better than cure: Addressing anti-vaccine conspiracy theories. J Appl Soc Psychol. 2017;47:459–69. - Cook J, Lewandowsky S, Ecker UKH. Neutralizing misinformation through inoculation: Exposing misleading argumentation techniques reduces their influence. PLoS One. 2017;12(5). - Mihailidis P, Viotty S. Spreadable Spectacle in Digital Culture: Civic Expression, Fake News, and the Role of Media Literacies in “Post-Fact” Society. Am Behav Sci. 2017;61(4):441 –454. |
| R2 | 23:3:1 What were the guidelines? | Examples provided (page 30, lines 621–622). |
| R2 | 23:3:11-12 "2.8 times less people were willing to share fake news following the treatment than before the treatment." - It is not clear what this statistic means and how it was identified. | The sentence has been clarified (page 30, 631–633). |
| R2 | 24:3:9-10 What are the flags? We assume they are materials in a study but this is not entirely clear. | The term has been corrected (page 31, lines 668–669). |
| R2 | 25:2:1-3 The authors discuss avoiding backfire effects, but research surrounding backfire effects is complicated. The current understanding is that backfire effects are not nearly as much of a concern as once thought - these recent findings should be reflected in this paragraph. (e.g., see Swire et al., 2020, DOI: 10.1016/j.jarmac.2020.06.006). | The debate around the backfire effects has been summarized in the paragraph (page 32, lines 689–697). Following citations were used:   - Swire-Thompson B, DeGutis J, Lazer D. Journal of Applied Research in Memory and Cognition Searching for the Backfire Effect: Measurement and Design Considerations. J Appl Res Mem Cogn. 2020;9(3):286–99. - Wood T, Porter E. The Elusive Backfire Effect: Mass Attitudes ’ Steadfast Factual Adherence. Polit Behav. 2019;41:135–63. - Nyhan B, Reifler J. When Corrections Fail: The Persistence of Political Misperceptions. Polit Behav. 2010;32:303–30. - Hart PS, Nisbet EC. Boomerang Effects in Science Communication: How Motivated Reasoning and Identity Cues Amplify Opinion Polarization About Climate Mitigation Policies. Communic Res. 2012;39(6):701–723. |
| R2 | 27:2:2 What is meant by a "common decision environment"? A definition here would be useful. | Sentence reworded (page 34, lines 759–762). |
