# Supplementary material for: Determinants of individuals’ belief in fake news: A scoping review determinants of belief in fake news
Source: PLoS One. 2021 Jun 24;16(6):e0253717. doi: 10.1371/journal.pone.0253717 (PMC8224890; doi:10.1371/journal.pone.0253717)
Supplement: Attachment — Submitted filename: Response to reviewers.docx [file pone.0253717.s002.docx]

**Rebuttal letter**

Dear Drs. Altay and Lewandowsky,

We are grateful for your continued contributions to the improvement of our work. The revised manuscript addresses each comment you have raised in the latest round of review. Further details on the changes we have made can be found in the table below. We hope that you will deem the resulting manuscript fit for publication.

Kind regards,

The authors.

| **Reviewer** | **Comment** | **Response** |
| --- | --- | --- |
| R1 | It's too bad that the very small scale of the fake news problem is not mentioned in the introduction (e.g. Allen et al. 2020) but the article is, I believe, good enough to be published as is. | A note with the suggested citation added to the introduction section (lines 53-54 (in the “Revised Manuscript with Track Changes” version)). |
| R2 | 165: I am not entirely clear why “cognitive science research on false memory recognition” would be “obviously irrelevant”? | This seems an unfortunate turn of phrase: Clearly, false memory recognition can be substantively related to perception of fake news. Our point here was that the original query captured a large number of such cognitive studies, all of which were ultimately categorized as not fitting the scope of our review. The sentence is corrected and now includes an example of an actually “obviously irrelevant” category of studies: research reports mentioning false-positive biochemical tests results, also abundantly present in the search output (line 207). |
|  | 173: Does “non-experimental” mean the authors excluded correlational studies? I would have thought most individual-differences research may involve correlational studies that do not include an experimental intervention. Perhaps the authors mean “non-empirical”? If they did exclude correlational studies I would be curious to know why. | Our definition of experiment has been purposefully lax, since we acknowledged the possibility that not all relevant studies could employ rigorous experimental design with random assignment and a control group. We therefore included investigations where researchers varied at least one of the elements of news consumption process: either a hypothesized factor of belief in fake news (both between or within subjects), or veracity of news used as a stimulus (within-subjects). Consequently, the studies included in our scope presented both causal and correlational evidence. With the selection process set up like this, the only kind of study that wouldn’t make it into our sample would be the one with correlational design where respondents were only exposed to fake news.  We have modified the text accordingly to make this point more conspicuous to the reader (lines 149–157). |
|  | 23?: The authors may be interested in DOI 10.3758/s13421-019-00948-y as another demonstration of social influences (although it is only indirectly related to fake news because the study compares pro- and anti-science blog posts). | Citation worked into the manuscript as appropriate (lines 397–399). |
|  | 387 Insert paragraph break before “As this…”. | Corrected (line 481). |
|  | 392-394 This sentence is ungrammatical. | Sentence broken in two and reworded (lines 484–487). |
|  | 504 “the Indian sample” pops out of nowhere—this deserves a bit more explanation. Why India? What can be learned from this? | Clarification added (line 619). |
|  | 579 “messages” should be plural? | Corrected (line 708). |
